# Shared Regulatory Pathways Reveal Novel Genetic Correlations Between Grip Strength and Neuromuscular Disorders

**DOI:** 10.3389/fgene.2020.00393

**Published:** 2020-04-24

**Authors:** Sreemol Gokuladhas, William Schierding, David Cameron-Smith, Melissa Wake, Emma L. Scotter, Justin O’Sullivan

**Affiliations:** ^1^Liggins Institute, The University of Auckland, Auckland, New Zealand; ^2^Singapore Institute for Clinical Sciences, Agency for Science, Technology and Research (A^∗^STAR), Singapore, Singapore; ^3^Murdoch Children’s Research Institute, The University of Melbourne, Parkville, VIC, Australia; ^4^Department of Pharmacology and Clinical Pharmacology, The University of Auckland, Auckland, New Zealand; ^5^Centre for Brain Research, The University of Auckland, Auckland, New Zealand

**Keywords:** neuromuscular disorders, myasthenia gravis, multiple sclerosis, amyotrophic lateral sclerosis, muscle weakness, axon guidance pathway

## Abstract

Muscle weakness is a common consequence of both aging (sarcopenia) and neuromuscular disorders (NMD). Whilst genome-wide association (GWA) studies have identified genetic variants associated with grip strength (GS; measure of muscle strength/weakness) and NMDs, including multiple sclerosis (MS), myasthenia gravis (MG) and amyotrophic lateral sclerosis (ALS), it is not known whether there are common mechanisms between these phenotypes. To examine this, we have integrated GS and NMD associated genetic variants (single nucleotide polymorphisms; SNPs) in a multimorbid analysis that leverages high-throughput chromatin interaction (Hi-C) data and expression quantitative trait loci data to identify target genes (i.e., SNP-mediated gene regulation). Biological pathways enriched by these genes were then identified using next-generation pathway enrichment analysis. Lastly, druggable genes were identified using drug gene interaction (DGI) database. We identified gene regulatory mechanisms associated with GS, MG, MS, and ALS. The SNPs associated with GS regulate a subset of genes that are also regulated by the SNPs of MS, MG, and ALS. Yet, we did not find any genes commonly regulated by all four phenotype associated SNPs. By contrast, we identified significant enrichment in three pathways (mTOR signaling, axon guidance, and alcoholism) that are commonly affected by the gene regulatory mechanisms associated with all four phenotypes. 13% of the genes we identified were known drug targets, and GS shares at least one druggable gene and pathway with each of the NMD phenotypes. We have identified significant biological overlaps between GS and NMD, demonstrating the potential for spatial genetic analysis to identify common mechanisms between potential multimorbid phenotypes. Collectively, our results form the foundation for a shift from a gene to a pathway-based approach to the rationale design of therapeutic interventions and treatments for NMD.

## Introduction

Age-associated changes in skeletal muscle structure and composition are influenced by various factors including genetics, physical inactivity, malnutrition, hormonal and environmental changes (Fried et al., 2001; Brook et al., 2016; Morley, 2017). Understanding the biological pathways that contribute to disease and age-associated muscle wasting is critical if we are to develop effective therapies. Grip strength (GS) is strongly correlated with age-related loss of muscle strength (sarcopenia) and therefore is used as a predictor of health-related quality of life (Sayer et al., 2006; Ho et al., 2019). Weakness and wasting of the body due to severe chronic illness (cachexia) is a primary characteristic in a number of chronic neuromuscular diseases (NMD), including myasthenia gravis (MG), amyotrophic lateral sclerosis (ALS), and multiple sclerosis (MS) (Al-Chalabi and Hardiman, 2013; Manca et al., 2016; Vinge et al., 2019). For all of these diseases, the primary disease diagnosis is complicated by reduced physical function, reduced tolerance for therapy, increased burden on the healthcare system, and increased mortality that comes with cachexia (Arthur et al., 2014). Numerous mechanisms have been suggested to be involved in NMD-related muscle weakness, including mitochondrial dysfunction (Nardin and Johns, 2001), reduced numbers of motor units (Kaya et al., 2013), and functional deficits at the neuromuscular junction (NMJ) (van der Pijl et al., 2016).

MG is an autoimmune disease caused by autoantibodies (Abs) binding to postsynaptic region and impairing the function of acetylcholine receptors (AChRs). Three main pathogenic mechanisms mediated by these Abs underlie muscle weakness in MG: (1) IgG Abs bind to complement factors leading to the formation of a membrane attack complex; (2) specific IgG cross-linking of AChRs increase endocytosis and degradation of AChRs; or (3) blockage of AChR ion channels by Abs prevents the binding of acetylcholine to AChR (Verschuuren et al., 2013; Paz and Barrantes, 2019). These represent three direct mechanisms between skeletal muscle and MG etiology.

ALS is a fatal motor neuron disease that causes progressive degeneration of upper and lower motor neurons in the brain and spinal cord leading to muscle weakness, paralysis and death. In ALS, skeletal muscle harbors the characteristic protein aggregates (composed principally of TDP-43) (Cykowski et al., 2018) whose burden in the central nervous system (CNS) predicts neurodegeneration (Brettschneider et al., 2014; Mackenzie and Neumann, 2016). In muscle, these TDP-43 protein aggregates may disrupt the transport of mRNAs essential for muscle fiber regeneration (Schmid et al., 2013; Vogler et al., 2018). A recent study also demonstrated that phosphorylated TDP-43 aggregates in skeletal and cardiac muscle are a marker of myogenic degeneration in ALS (Mori et al., 2019). Together these findings support a direct links between skeletal muscle and ALS etiology.

MS is an autoimmune-mediated demyelinating disease of CNS. A cascade of events involving autoreactive T cells, B cells, microglia, astrocytes induce demyelination, inflammation and neurodegeneration in MS (Dendrou et al., 2015). Changes in the characteristics of skeletal muscle are observed in MS such as altered muscle function in MS includes impaired excitation-contraction coupling (Sharma et al., 1995), reduced oxidative capacity of the skeletal muscle (Harp et al., 2016), slowing of muscle contraction (Kent-Braun et al., 1994), and decreased cross-sectional area of type I and type II muscle fibers (Kent-Braun et al., 1997). However, pathological mechanisms that cause muscle weakness in MS have yet to be determined.

To date, there has been only one genome-wide association (GWA) study on the genetic features of cachexia, and only for body mass loss in COPD and cancer (McDonald et al., 2017). However, GWA studies have identified genetic variations [i.e., single nucleotide polymorphisms (SNPs)] associated with GS (Willems et al., 2017), MG (Renton et al., 2015), MS (Patsopoulos and de Bakker, 2011) and ALS (Fogh et al., 2014). These studies employed case-control approaches to identify the SNPs that are associated with these disorders. Most of these SNPs are located in the non-protein coding (i.e., regulatory) regions of the genome (Jo and Choi, 2015). The rarity of SNPs within coding sequences increases the difficulty of understanding how they function to impact on phenotype. One putative function is through alterations in regulatory regions (e.g., enhancers) through alteration of allele-specific long-range DNA contacts. Thus, the regulatory regions can be separated from their target genes by great distances (Pomerantz et al., 2009). Integrating the three-dimensional organization of the genome, captured by proximity ligation (e.g., Hi-C) (Belton et al., 2012), with functional data (e.g., eQTLs) can identify the targets of these long-range regulatory interactions (Fadason et al., 2018).

Co-occurrences of MG:MS (Somer et al., 1989; Isbister et al., 2003), MG:ALS (del Mar Amador et al., 2016; de Pasqua et al., 2017; Tai et al., 2017) and MS:ALS (Etemadifar et al., 2012; Guennoc et al., 2018) in patients and families have been reported, consistent with an etiological relationship or multimorbidity between these diseases. We hypothesize that GS, MG, MS, and ALS share common biological pathways consistent with their common age-associated and/or disease-associated muscle wasting and weakness. In the present study, we identify the genes that are regulated by GS-, MG-, MS-, and ALS-associated SNPs using high throughput chromatin interaction data (Hi-C) from 8 immortalized cell-lines and psoas muscle tissue (i.e., representative of the genomic contacts seen across cell types or only in skeletal muscle, respectively). Combining these spatial interaction data with functional eQTL data, we identify gene regulatory (i.e., eQTL-eGene) interactions associated with each phenotype. Subsequent pathway analyses identified the biological pathways associated with these phenotypes. Collectively, our analysis identifies numerous genetic and biological pathway overlaps between neuromuscular diseases (MS, MG, and ALS) and GS, including three pathways that overlap all four. This suggests common and distinct genetic mechanisms underlying muscle weakness associated with age and disease.

## Materials and Methods

### Identification of Functionally Significant Spatial Regulatory Interactions

SNPs associated with GS were identified from the published literature (Supplementary Table 1a). MG, MS, and ALS associated SNPs were obtained from GWAS catalog^1^ (Supplementary Tables 1b–d). Using the CoDeS3D algorithm (Fadason et al., 2017), we mapped spatial regulatory connections between each phenotype-associated SNP and one or more gene coding regions (GENCODE release 19). For each SNP-gene pair, spatial contacts were identified from existing Hi-C (Belton et al., 2012) chromatin contact data derived from eight immortalized cell lines representing human germ layer lineages (GM12878, IMR90, HMEC, NHEK, K562, HUVEC, HeLa, and KBM7) (Rao et al., 2014) and one tissue (Psoas muscle) (Schmitt et al., 2016) (Supplementary Table 2). The SNP-gene pairs that were identified using the Hi-C contact data were queried against the Genotype-Tissue Expression (GTEx) database, which contains data on human gene expression and genetic variation in 48 human tissues (Carithers and Moore, 2015), to identify SNPs (expression Quantitative Trait Loci, eQTLs) that are associated with expression changes of the partner genes (eGenes). The resultant eQTL-eGene associations were corrected for multiple testing, using the Benjamini-Hochberg False Discovery Rate (FDR) correction (Fadason et al., 2017). Chromosome positions of eQTLs and eGenes were annotated according to Human reference genome GRCh37/hg19 assembly.

### Annotation of Significant eGenes

Gene ontology enrichment analysis using the Gene Ontology (GO) Consortium web-based knowledgebase (Ashburner et al., 2000; Carbon et al., 2019)^2^ identified the biological functions that were overrepresented by the eGenes shared between GS and NMD associated eQTLs. Overrepresentation analysis was performed using PANTHER and the reference set of all *Homo sapiens* protein-coding genes was used as a background set. *P*-values were subjected to Bonferroni multiple testing correction with FDR *p* < 0.05 being deemed significant.

### Identification of Enriched Pathways

We performed pathway enrichment analysis (iPathwayGuide^3^) to identify pathways enriched for eGenes from GS, MG. MS and ALS. Analyses were performed using default parameters as described by Ahsan and Draghici (Ahsan and Drăghici, 2017). The overrepresented pathways with FDR *p* < 0.05 was used for further analysis.

### Identification and Validation of Overlapping eGenes Across Phenotypes

R studio (Version 1.2) was used to compare eGene lists for each phenotype to identify intersections between all possible phenotype combinations. To test if gene overlaps were non-random, we performed bootstrap analysis using 10000 randomly chosen sets of genes [from a background set of all significant eGenes (34,461 eGenes) from GTEx (Version 8)] of the same size as the number of eGenes for each phenotype.

### Retrieving Information About Druggability of eGenes

The druggable eGenes were identified by querying all eGenes against the Drug Gene Interaction database (DGIdb^4^) which integrates information on gene druggability and drug-gene interactions from 30 different sources (Griffith et al., 2013; Cotto et al., 2018).

## Results

### SNPs Associated With Generalized Muscle Weakness (GS) and Neuromuscular Disorders (MG, MS, and ALS) Form an Overlapping Functional Regulatory Network

**FIGURE 1:**
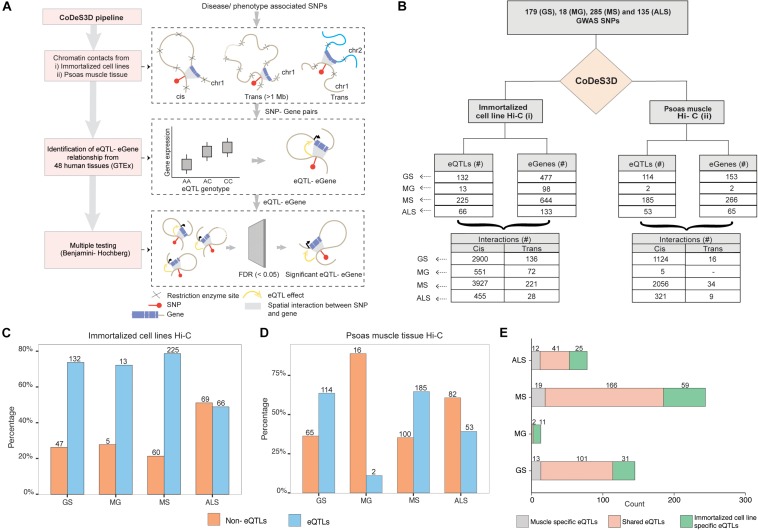
GWAS SNPs associated with GS, MG, MS, and ALS mark functional regulatory loci. **(A)** CoDes3D workflow: The regulatory SNP-gene interactions (cis and trans) were identified from existing Hi-C datasets [i.e., immortalized cell lines dataset from Rao et al. (2014) and psoas muscle tissue dataset from Schmitt et al. (2016)]. The SNP-gene pairs were tested against the GTEx database to identify eQTL effects on the spatially connected genes. Those eQTL-eGenes with FDR < 0.05 were considered significant. **(B)** Summary statistics of the regulatory interactions (spatial eQTLs) identified between phenotype (GS, MG, MS, and ALS)-associated SNPs and eGenes. eQTLs were identified by integrating spatial information from Hi-C contact maps for immortalized cell lines (Rao et al.; left) or psoas muscle (Schmitt et al.; right) with eQTL data from GTEx (FDR < 0.05). Cis eQTLs are located ≤1 Mb from the target eGene. Trans eQTLs are located >1 Mb from the target eGene or on a different chromosome. **(C,D)**. The proportion of eQTL: non-eQTL SNPs identified using **(C)** the immortalized cell line Hi-C datasets (Rao et al., 2014), or **(D)**. the psoas muscle Hi-C dataset (Schmitt et al., 2016) **(E)**. Numbers of eQTLs specific to immortalized cell line and psoas muscle, or both Hi-C datasets.

We identified 179, 18, 285, and 135 SNPs that were associated with GS, MG, MS, and, ALS, respectively, at a suggestive level of significance (*p* ≤ 5 × 10^–6^; Supplementary Table 1). Phenoytpe associated SNP sets were analyzed through the CoDeS3D (Fadason et al., 2017) pipeline. The pipeline uses chromatin interaction (Hi-C) data from immortalized cell lines (Rao et al., 2014) to identify all gene coding regions that are physically interacting with the phenotype associated SNPs (SNP-gene pairs). The GTEx database was then used to test for regulatory relationship between the identified SNP-gene pairs using the eQTL-eGene associations within 48 human tissues. The significance of each association was tested using the Benjamini-Hochberg FDR correction method (Figure 1A).

Despite the common muscle weakness phenotype presented by these disorders, there were no common SNPs shared between the sets of disease associated SNPs for each of these disorders. We identified SNP-gene spatial connections associated with eQTLs for GS (*n* = 179), MG (*n* = 18), MS (*n* = 285), and ALS (*n* = 135) SNPs (Figure 1B). Approximately 95% of the spatial eQTL-eGene regulatory interactions identified across each phenotype occur between proximal (cis; ≤ 1 Mb between the SNP and the gene) rather than distal (trans; >1 Mb between the SNP and gene, or the SNP and gene are located on different chromosomes) regions (Figure 1B and Supplementary Table 3). We then tested for phenotype-specific enrichment in the proportion of eQTLs versus non-eQTLs. While nearly 80% of the SNPs associated with GS, MG, and MS are eQTLs, less than 50% of ALS-associated SNPs are eQTLs (Figure 1C).

We hypothesized that the emergent genome organization in muscle would be more relevant to gene regulation in understanding the biological underpinnings of sarcopenia and NMD. Therefore, we repeated the CoDeS3D analysis using genomic organization data captured in psoas muscle tissue (Schmitt et al., 2016) (Figure 1B (right); Supplementary Table 4). We identified phenotype-specific enrichment of eQTLs in the psoas muscle Hi-C dataset (Figure 1D). Furthermore, consistent with our hypothesis, and despite the immortalized cell line Hi-C dataset being nearly 10 times larger, a considerable number of the identified regulatory interactions were specific to muscle (Figure 1E, Supplementary Figure 1). However, we did not identify any eQTLs specific to psoas muscle using SNPs associated with MG. This suggests that MG-associated SNPs do not make a major contribution to the regulatory networks within skeletal muscle.

### Shared eGenes Between GS, MG, MS, and ALS Suggest Common Genetic Mechanisms

**FIGURE 2:**
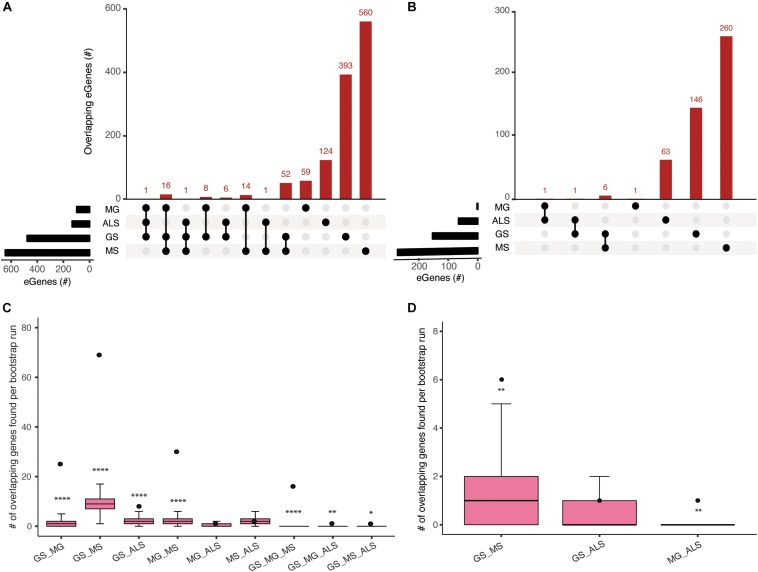
Comparisons of spatial regulatory interactions identified eGenes that are shared between GS, MG, MS, and ALS. Overlap between eGene sets for GS, MG, MS, and ALS identified using **(A)** immortalized cell line Hi-C datasets, or **(B)** the Psoas muscle Hi-C dataset. The total number of eGenes contained in each individual set is represented as horizontal bars (black). The dot matrix denotes phenotype overlap, where the line connecting dots indicates the sets being compared. **(C)** and **(D)** Boxplots showing the mean and range of bootstrapping values (*n* = 10000) for overlaps between randomly selected gene sets (from a set of 34,461 genes) to validate overlaps from **(C)** the immortalized cell line Hi-C dataset, or **(D)** the Psoas muscle Hi-C dataset. The black point represents the observed number of eGene overlaps between the stated phenotypes. ****p-value <1 × 10^– 4^, **p-value <0.01, *p-value <0.05.

The clinical manifestations, diagnoses, and treatments of MG, MS, and ALS differ. However, despite having no overlapping SNPs, our analysis identified 89 shared eGenes between these phenotypes and generalized muscle weakness (GS; Figure 2 and Supplementary Table 5). The degree of eGene overlap between phenotypes was dependent upon the Hi-C datasets that were used to identify the eQTLs (compare Figures 2A,B). Bootstrapping (10000 test intersections) with all identified eGenes (see methods) confirmed that the shared eGene overlaps identified using spatial SNP-gene sets captured within the immortalized cell lines were significantly greater than expected by chance (Figure 2C). Similarly, comparisons of eGenes identified using psoas muscle Hi-C data identified significant overlaps between GS:MS (Figure 2D). To further assess the significance of observed overlaps from both immortalized cell line and muscle datasets, we performed bootstrapping analysis with another gene background set consisting only of those genes (*n* = 18452) that were captured having spatial contacts with any of the four phenotype associated SNP sets. The results were consistent with what we saw using the all eGenes background set. However, the overlap between GS:MS:ALS was no longer significant for the immortalized cell line dataset using only those genes with a spatial connection (Supplementary Figure 2).

We identified 30 shared eGenes (mainly HLA loci genes) between MG and MS eQTLs; *ERMP1* and *SLC25A12* were regulated by MS and ALS eQTLs; and *C18orf8* was regulated by MG and ALS eQTLs (Supplementary Table 5). The 25 eGenes in common between GS and MG were overrepresented in immune system-related processes, including antigen processing and presentation, interferon-gamma-mediated signaling, T-cell receptor signaling and adaptive immune responses (Supplementary Table 6a). Similarly, ontological analysis of the 69 eGenes in common between GS and MS identified enrichment for immune system functions (Supplementary Table 6b).

Genes in the HLA-region were overrepresented in the overlapping eGene sets. We hypothesized that the observed overlaps in the HLA region were not due to the effect of Linkage disequilibrium (LD) between eQTLs in that region. We derived the LDmatrix between all four phenotype associated SNPs located across chromosome 6 using LDlink^5^. We identified one eQTL (rs9270986) associated with MG in LD (R^2^ = 0.96) with a single eQTL (rs3129889) associated with MS. Consistent with our hypothesis, we did not find any other eQTLs in strong LD (R^2^ > 0.8) (Supplementary Figure 3). Collectively, our observation of shared eGenes is consistent with a partially shared genetic etiology underlying the development of muscle weakness between sarcopenia and NMD.

### Overlapping GS, MG, MS, and ALS Molecular Pathways Reveal Putative Therapeutic Targets

**FIGURE 3:**
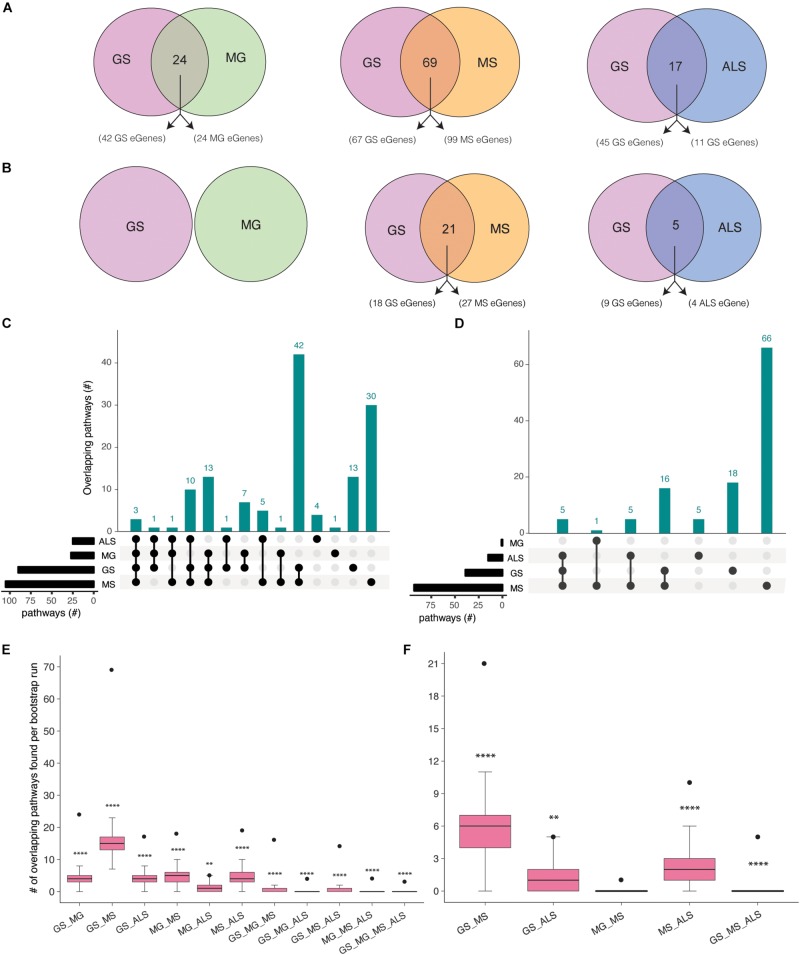
Co-occurrence of condition-specific eGenes within biological pathways is associated with comorbidity between GS, MG, MS, and ALS. Overlap of biological pathways that contain phenotype-associated eGenes identified using chromatin interactions from **(A)** immortalized cell lines, or **(B)** the psoas muscle. Numbers of overlapping pathways enriched with eGenes from GS, MG, MS, and ALS identified from **(C)** immortalized cell line Hi-C dataset and **(D)** muscle tissue Hi-C dataset are plotted on the *y*-axis. The total number of pathways impacted by each set of eGenes is represented as horizontal bars (black). **(E, F)**. Boxplots showing the mean and range of bootstrapping values (*n* = 10000) for overlaps between randomly selected gene pathways (from all KEGG pathways) to validate overlaps from **(E)**. the immortalized cell line Hi-C dataset, or **(F)** the muscle Hi-C dataset. The black point represents the observed number of eGene overlaps between the stated phenotypes. ****p-value <1 × 10^– 4^, **p-value <0.01, *p-value <0.05.

Gradual muscle wasting and weakness is a comorbidity that we hypothesized would be reflected in shared biological pathways between MS, MG, ALS, and GS. Using iPathwayGuide, we identified considerable number of pathways (Supplementary Figure 4) and overlapping pathways between GS and NMD associated eGenes (Figures 3A,B). For example, GS (22 out of 42) and MG (16 out of 24) eGenes (immortalized cell line Hi-C dataset) are highly enriched for immune system and related pathways (Supplementary Figure 5 and Supplementary Table 7a). Similarly, GS and MS eGenes are enriched for immune system, signal transduction, nervous system, and endocrine system-associated pathways (Supplementary Figure 6 and Supplementary Table 7b). eGenes for GS and ALS co-occurred within signal transduction, substance dependence, and cell motility pathways (Supplementary Figure 7 and Supplementary Table 7c).

Shared eGenes explained some of the observed co-occurrence in pathways; >50% of the shared pathways observed for GS:MG and GS:MS (immortalized cell line Hi-C datasets) contained both shared and condition-specific eGenes. By contrast, 99% of shared pathways identified for GS and ALS were due to co-occurrence of condition-specific eGenes. Similarly, all of the overlapping pathways (GS:MS and GS:ALS) identified using eGenes that were derived from the psoas muscle dataset were due to the co-occurrence of condition-specific eGenes (Supplementary Tables 7d,e). Thus, pathway overlap was largely due to the co-occurrence of condition-specific eGenes within the same functional networks.

**FIGURE 4:**
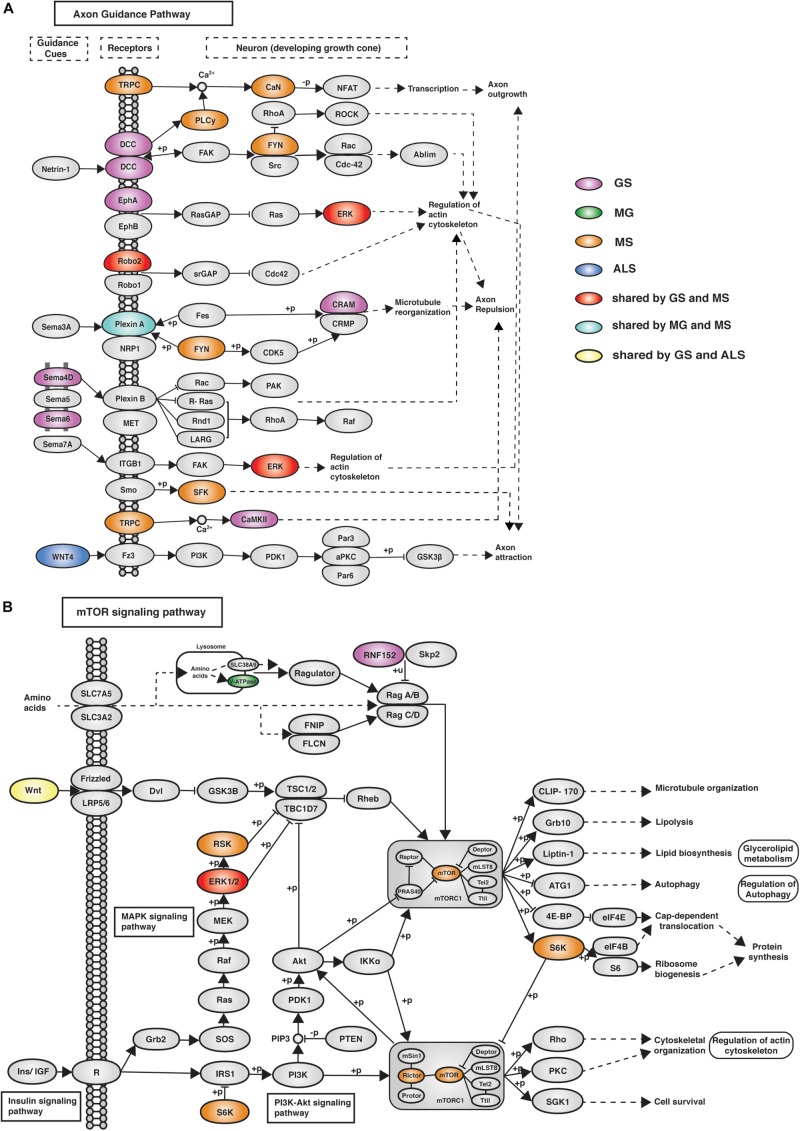
eGenes from all four phenotypes (GS, MG, MS, and ALS) co-occurred within the axon guidance and mTOR signaling pathways. **(A)** The axon guidance pathway is involved in neural development and defects are associated with neuronal disorders. **(B)** The mTOR signaling pathway controls cellular processes including: cell growth, protein synthesis, cytoskeletal organization, and autophagy (Laplante and Sabatini, 2009). GS-, MG-, MS-, and ALS-associated eQTLs spatially regulate multiple eGenes that encode proteins involved in these pathways. Notably, proteins encoded by ALS eGenes are upstream regulators in both pathways.

eGenes associated with NMD also co-occur in shared pathways, consistent with the existence of multimorbidity between these conditions (Supplementary Table 8). Notably, while there were no eGenes that were shared by all four phenotypes (Figure 2A), there were three pathways (i.e., axon guidance, alcoholism and the mTOR signaling pathway) that contained co-occurring eGenes from all four conditions (Figure 3C). For example, the axon guidance pathway contained eGenes that were regulated by genetic variants from one or multiple phenotypes (Figure 4A). Similar patterns were observed in both the mTOR signaling (Figure 4B) and alcoholism pathways (Supplementary Figure 8).

The eGenes associated with GS, MG, MS, and ALS that were identified using the psoas muscle dataset also co-occurred in biological pathways (Figure 3D). The shared biological pathways that contained eGenes identified using the psoas muscle Hi-C dataset represented a subset of overlapping pathways identified using the eGenes from the immortalized cell line Hi-C dataset (Supplementary Table 8b).

To test the significance of identified pathway overlaps, we performed bootstrap analysis using the list all KEGG pathways (*n* = 536) as a background set. Ten thousand random samples were generated of the same size as the number of phenotype associated pathways. Bootstrap analysis reinforced that all pathway overlaps identified (using eGenes identified from immortalized Hi-C dataset) between phenotypes were statistically more significant than expected by chance (Figure 3E). Similarly, bootstrap analysis confirmed that the pathway overlaps identified using eGenes from the muscle Hi-C dataset were not due to chance (Figure 3F). However, the pathway overlap identified between MG and MS was not significant (*P*-value = 0.1638; Figure 3F). To be more conservative, we also performed the bootstrap analysis using the list of all pathways containing eGenes identified for GS, MG, MS and ALS (*n* = 267 for immortalized cell line dataset and *n* = 247 for the muscle Hi-C dataset). Again, similar results were observed from both analyses (Supplementary Figure 9) consistent with the overlaps for all combinations, except MG:ALS, occurring more frequently than expected by chance.

### Overlapping, Treatable Biological Pathways Are Shared Drug Targets for GS, MG, MS, and ALS

**FIGURE 5:**
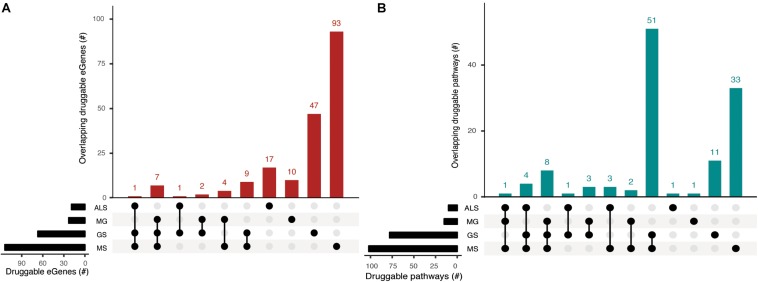
Phenotypes share druggable eGenes and druggable pathways. Overlapping **(A)** druggable eGenes and **(B)** pathways they occur in, as identified from the immortalized cell line Hi-C dataset. The number of druggable eGenes identified for each phenotype (left) and the number of druggable pathways identified for each phenotype (right) are represented as horizontal bars. The matrix denotes the intersection between sets under comparison.

eGenes that encode druggable proteins may have therapeutic value, especially as targets for drug repurposing or for the identification of potential targets for shared drug morbidity. Using the drug-gene interaction database (DGIdb), we determined that 13% of the eGenes we identified are druggable (Supplementary Tables 9, 10). Notably, GS shares at least one druggable eGene and pathway with each of the NMD phenotypes (Figure 5, Supplementary Tables 11a,b). While there were no druggable eGenes between ALS and MG, there was one druggable pathway (i.e., cytokine-cytokine receptor interaction pathway) that was shared between them (Supplementary Table 11c).

We identified entirely unique sets of druggable eGenes for GS (4 eGenes), MS (18 eGenes) and ALS (1 eGene) from the psoas muscle that were not found in immortalized cell lines (Supplementary Table 9). Three out of four GS druggable eGenes co-occur within the neuroactive ligand-receptor interaction pathway. Nine out of eighteen MS druggable eGenes were involved in 25 different pathways, predominantly in signal transduction pathways (Supplementary Table 11d). From the psoas muscle Hi-C dataset we identified unique druggable eGenes (e.g., GRID1, TNFRSF1A, STAT4) which represent potential therapeutic targets for treating the muscle wasting/weakness symptoms associated with NMD and sarcopenia.

## Discussion

This is the first report of shared biological mechanisms between genetic variants associated with the generalized age-associated decline of muscle strength (as measured by hand GS) and muscle weakness/wasting caused by neuromuscular disorders (i.e., MG, MS, and ALS). The existence of shared eGenes, whose expression correlates with condition-associated SNPs, is consistent with subtle changes within combinatorial gene regulatory mechanisms contributing to the risk of generalized aspects of muscle weakness. Moreover, the observation of the co-occurrence of condition-specific eGenes within shared biological pathways provides insights into the mechanisms associated with the risk of development of generalized age- and disease-associated muscle weakness.

Altered concentrations of circulating immune markers and heightened immune responses have been shown to lead to reduced muscle protein synthesis (Toth et al., 2005). We identified eGenes that contribute to immune system-related functions and whose expression levels were correlated with genetic variants associated with GS and MG or MS. The co-occurrence of eGenes (e.g., HLA loci genes, *C2*, *TAP2*, *MICB*) specific to each of the phenotypes within immune system-related pathways is consistent with these pathways having a significant role in the development of age and disease-related muscle weakness. Similarly, we determined that the expression level of the *SLC25A12* gene is associated with rs2044469 (GS), rs4953911, and rs882300 (MS) and, rs1400816 (ALS). Notably, alterations to *SLC25A12* expression affect mitochondrial structure, function and biogenesis and are associated with age-related muscle dysfunction (Liu et al., 2013). Collectively, these observations highlight the complex interrelationships between phenotypes, highlighting the potential contribution of changes to genomic elements that contribute to the combinatorial regulation of genes that affect normal muscle function and may thus contribute to the severity of neuromuscular diseases (Fenichel, 1966; Pansarasa et al., 2014; Wens et al., 2014; Loeffler et al., 2016).

The mTOR signaling pathway (hsa04150), axon guidance (hsa04360) and alcoholism (hsa05034) pathways contain eGenes whose regulation is associated with genetic variants from each of the four phenotypes. The axon guidance pathway guides axon outgrowth and plays a pivotal role in forming a properly functional nervous system (Chisholm et al., 2016). Axon guidance proteins involved in this pathway are responsible for neural circuit development and the changes in the expression and function of these proteins can cause neurological diseases. For example, increased expression of Sema3A and EPHA4 causes de-adhesion of neuromuscular junctions, leading to muscle denervation in ALS (Van Battum et al., 2015). Changes in the mTOR signaling pathway (e.g., the loss of mTOR in skeletal muscle) or alterations to mTOR-mediated processes can contribute to myopathy and other skeletal muscle pathologies (Risson et al., 2009). The mTOR protein coding gene expression correlates with the MS-associated variant rs3748817. Similarly, the overall function of the mTOR pathway is likely affected by alterations to RNF152, V-ATPase and WNT, which are eGenes for GS-, MG-, and ALS-specific variants, respectively. Superficially the co-occurrence of eGenes within the KEGG-defined alcoholism pathway would appear to be inconsistent with our hypothesis. However, this pathway involves dopamine release, PKA signaling and activation of CREB-mediated gene expression. CREB activation promotes regeneration in instances of muscle damage (Stewart et al., 2011). Collectively, these findings are consistent with these pathways contributing core functions that mechanistically contribute to the maintenance and development of muscle strength.

We identified druggable eGenes and pathways for the MG, MS, ALS, and GS phenotypes. Crucially, the overlapping druggable pathways are not simply the result of the presence of shared druggable eGenes. For instance, we identified co-occurring druggable eGenes (i.e., MG: *LTA*; and ALS: *CX3CR1*) within the cytokine-cytokine receptor interaction pathway. The co-occurrence of shared and phenotype-specific eGenes within these pathway overlaps informs on potential pharmacological side-effects associated with therapeutic treatment (Soon et al., 2009). For example, vandetanib inhibits EPHA6 (receptor protein kinase) in the axon signaling pathway. However, in the presence of genetic variants that affect other genes within the axon signaling pathway (e.g., MS-associated eGene ERK), EPHA6 disruption could propagate through the axon signaling pathway to trigger the risk pathway for MS.

Overall, the SNP-gene pairs were observed across most of the Hi-C datasets used in this study. This is likely due to the conservation of topologically associated domains (TADs) across cell types and lineages (Dixon et al., 2012; Rowley et al., 2017). However, a subset of SNP-gene interactions was only captured in the psoas muscle Hi-C data set, suggesting that gene regulatory interactions may be tissue specific in nature. Crucially, the biological pathways that contained co-occurring eGenes, which were specific to psoas muscle, represented a subset of the pathways identified from the immortalized cell line Hi-C datasets. Notably, none of the biological pathways containing co-occurring eGenes, identified from the psoas muscle Hi-C dataset, contained shared eGenes. For example, the Ras signaling pathway (required for the differentiation of slow muscle fibers by inducing slow motor neurons) (Murgia et al., 2000) contains specific eGenes associated with: GS - *TGFA, RASGRF1*; MS- *RASGRP1, MAPK3, MAPK1, NFKB1, PLCG1, FLT1*; and ALS- *MRAS, TIAM1, FGF12*. Collectively, we contend that these observations reflect the tissue-specific impacts of the genetic variants and functional regulatory networks.

There are alternative explanations for the identification of more eGenes using Hi-C data from the immortalized cell lines (Rao et al., 2014) than from the psoas muscle (Schmitt et al., 2016). The simplest explanation is that the chromatin interaction patterns emerge from the underlying nuclear functions and thus represent the different transcriptional networks in the pluripotent and differentiated cells (Hawkins et al., 2010). Secondly, there is a strong linear relationship between the number of samples in GTEx and the number of eQTLs that are identified. Once saturated this relationship should reach an asymptote. Therefore, it remains possible that the tissue-specific pattern we are observing for the eGenes is due to under-sampling. Thirdly, cell-type-specific patterns of gene expression (Getzenberg, 1994; Dezsö et al., 2008) may be lost due to tissue averaging. Fourthly, variation in the experimental Hi-C protocols used to capture the genome organization (Rao et al., 2014; Schmitt et al., 2016) may have resulted in false negatives in the psoas muscle data set. Despite these limitations, the co-occurrence of eGenes within biological pathways – in the presence of limited numbers of shared eGenes – indicates the utility of this approach for the identification of pathways underlying phenotype-specific characteristics.

In conclusion, we have identified the existence of common genetic mechanisms by which neuromuscular diseases and aging cause muscle weakness and wasting. The shared gene regulatory mechanisms and the shared pathways identified between the phenotypes represent novel therapeutic targets and highlight possible mechanisms for drug side-effects and other complications.

## Code Availability

CoDeS3D pipeline is available at https://github.com/Genome3d/codes3d-v1. R and python scripts used for data cleaning and generating figures are available in figshare with the identifier 10.17608/k6.auckland.9795350. R Studio version 1.2.1335 was used for all R scripts. Python version 2.7.15 was used for all the python scripts.

## Data Availability Statement

All datasets generated for this study are included in the article /Supplementary Material.

## Ethics Statement

Written informed consent was obtained from the individual(s) for the publication of any potentially identifiable images or data included in this manuscript.

## Author Contributions

SG performed analysis, interpreted the data and wrote the manuscript. WS contributed to the data interpretation and commented on the manuscript. MW commented on the manuscript. DC-S commented on the manuscript. ES contributed to the data interpretation and commented on the manuscript. JO’S directed the study, co-wrote the manuscript, contributed to data interpretation and commented on the manuscript. JO’S is a guarantor for this manuscript.

## Conflict of Interest

The authors declare that the research was conducted in the absence of any commercial or financial relationships that could be construed as a potential conflict of interest.

## Footnotes

1 https://www.ebi.ac.uk/gwas, January 20, 2019

2 http://geneontology.org/, February 02, 2019

3 https://advaitabio.com/ipathwayguide/

4 http://www.dgidb.org/

5 https://ldlink.nci.nih.gov/
