# Supplementary material for: Shared Regulatory Pathways Reveal Novel Genetic Correlations Between Grip Strength and Neuromuscular Disorders
Source: Front Genet. 2020 Apr 24;11:393. doi: 10.3389/fgene.2020.00393 (PMC7194178; doi:10.3389/fgene.2020.00393)
Supplement: Supplementary file 1 [file Data_Sheet_1.docx]

***Supplementary Material***

1. **Supplementary Data**

Supplementary Table 1 is available in figshare with the identifier <https://doi.org/10.17608/k6.auckland.9789269>.

Supplementary Table 2 is available in figshare with the identifier <https://doi.org/10.17608/k6.auckland.9789299>.

Supplementary Table 3 is available in figshare with the identifier <https://doi.org/10.17608/k6.auckland.9789314>.

Supplementary Table 4 is available in figshare with the identifier <https://doi.org/10.17608/k6.auckland.9789416>.

Supplementary Table 5 is available in figshare with the identifier <https://doi.org/10.17608/k6.auckland.9789425>.

Supplementary Table 6 is available in figshare with the identifier <https://doi.org/10.17608/k6.auckland.9789440>.

Supplementary Table 7 is available in figshare with the identifier <https://doi.org/10.17608/k6.auckland.9789446>.

Supplementary Table 8 is available in figshare with the identifier <https://doi.org/10.17608/k6.auckland.9789458>.

Supplementary Table 9 is available in figshare with the identifier <https://doi.org/10.17608/k6.auckland.9789467>.

Supplementary Table 10 is available in figshare with the identifier <https://doi.org/10.17608/k6.auckland.9790688>.

Supplementary Table 11 is available in figshare with the identifier <https://doi.org/10.17608/k6.auckland.9790709>.

**Human genome build hg19 (GRChr37) was downloaded from** [**http://ftp.ensembl.org/pub/release-75/fasta/homo_sapiens/**](http://ftp.ensembl.org/pub/release-75/fasta/homo_sapiens/)**.**

**SNP annotations (human genome, build hg19) were obtained from** [**https://ftp.ncbi.nih.gov/snp/organisms/human_9606_b151_GRCh37p13/**](https://ftp.ncbi.nih.gov/snp/organisms/human_9606_b151_GRCh37p13/)**.**

**Gene annotations were downloaded from** [**http://www.gtexportal.org/static/datasets/gtex_analysis_v7/reference/gencode.v19.genes.v7.patched_contigs.gtf.gz**](http://www.gtexportal.org/static/datasets/gtex_analysis_v7/reference/gencode.v19.genes.v7.patched_contigs.gtf.gz)**.**

**
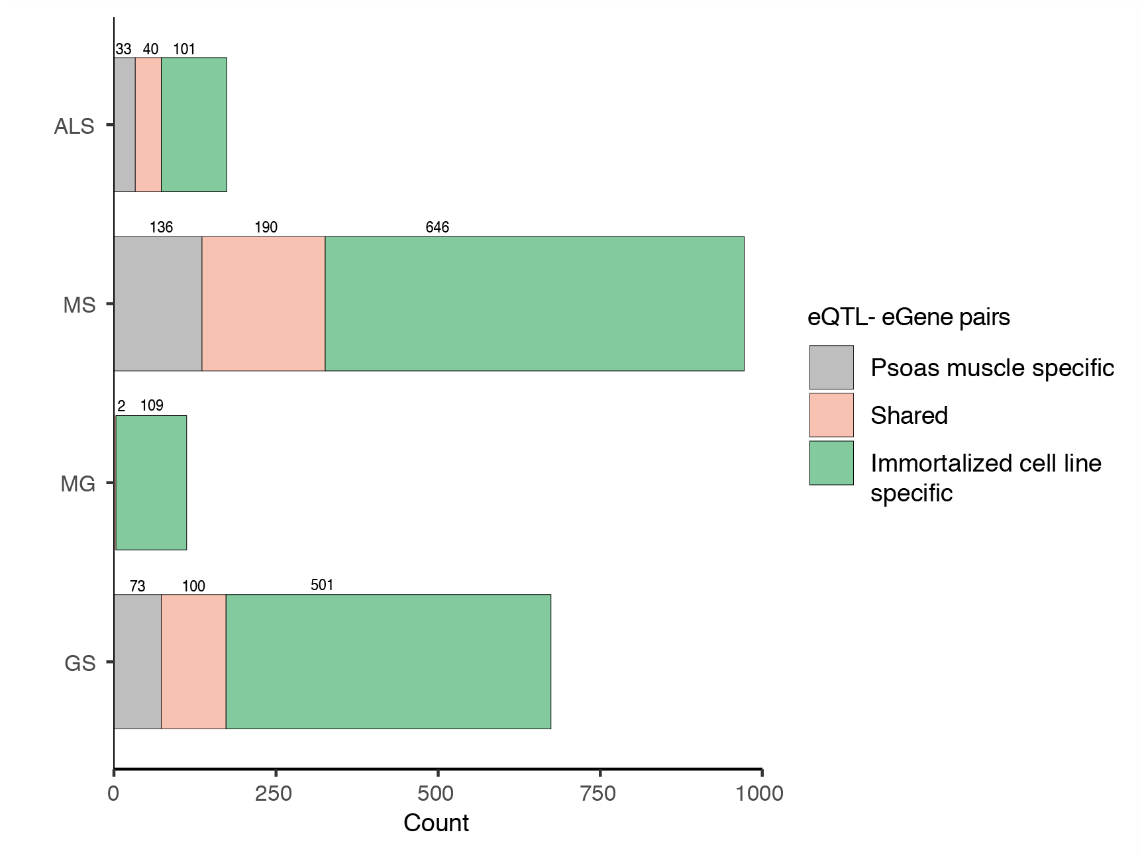
**

**Supplementary Figure 1.** eQTL-eGene pairs identified from immortalized cell line and psoas muscle Hi-C dataset. In all four phenotypes studied, more eQTL-eGene interactions were identified in the immortalized cell line Hi-C dataset, while the small number of interactions identified in the psoas muscle Hi-C dataset might represent tissue-specific chromatin interactions.

**Supplementary Figure 2.** eGene overlaps identified between all four phenotypes from both Hi-C datasets are statistically significant. Boxplots showing the mean and range of bootstrapping values (n=10000) for overlaps between randomly selected gene sets (from a set of 18,452 genes) to validate overlaps from the immortalized cell line Hi-C dataset (left) or the muscle Hi-C dataset (right). The black point represents the observed number of eGene overlaps between the stated phenotypes. **** p-value < 1x10^-4^ ** p-value < 0.01 *p-value <0.05

**Supplementary Figure 3: The majority of the** GS, MG and MS associated eQTLs located on chromosome 6 are not in LD. Linkage Disequilibrium (LD) plot of GS, MG and MS associated eQTLs on chromosome 6 amongst people with Western European ancestry (CEU). Each square represents the LD (R^2^) value between the two compared eQTLs. The weak LD (R^2^ < 0.8) indicates that the eQTLs are not frequently inherited together.


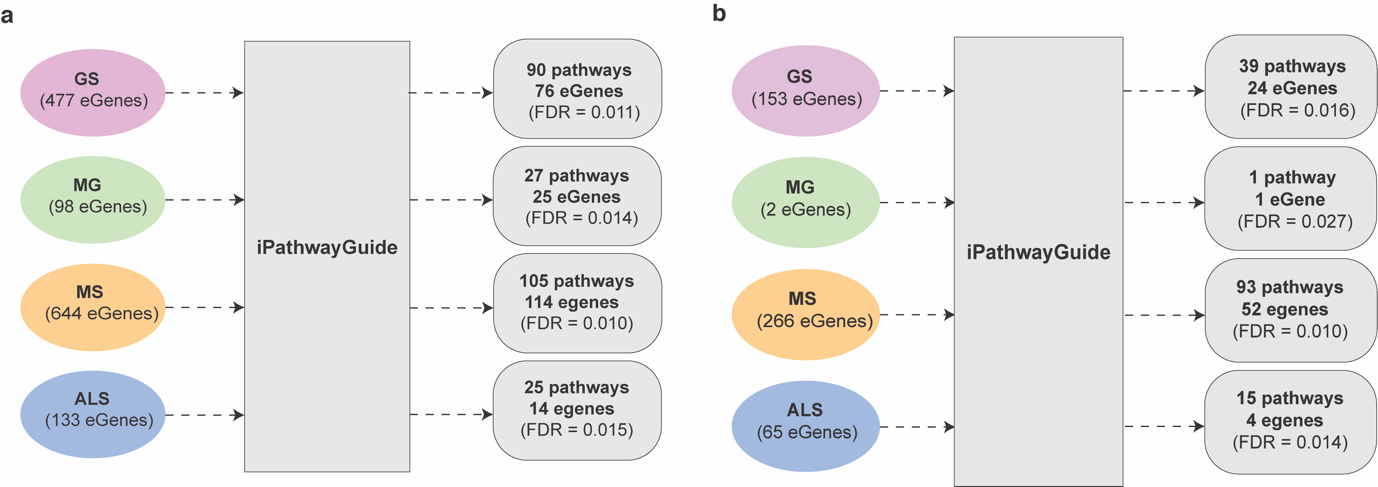


**Supplementary Figure 4.** GS, MG, MS and ALS associated eGenes are enriched in biological pathways. iPathwayGuide analysis of significant eGenes associated with GS, MG, MS and ALS from **a.** immortalised cell line and **b.** muscle Hi-C datasets identified biological pathways enriched by them with FDR < 0.05. Number of pathways and the total number of eGenes overrepresented in these pathways are provided on the right of **a** and **b**.

**
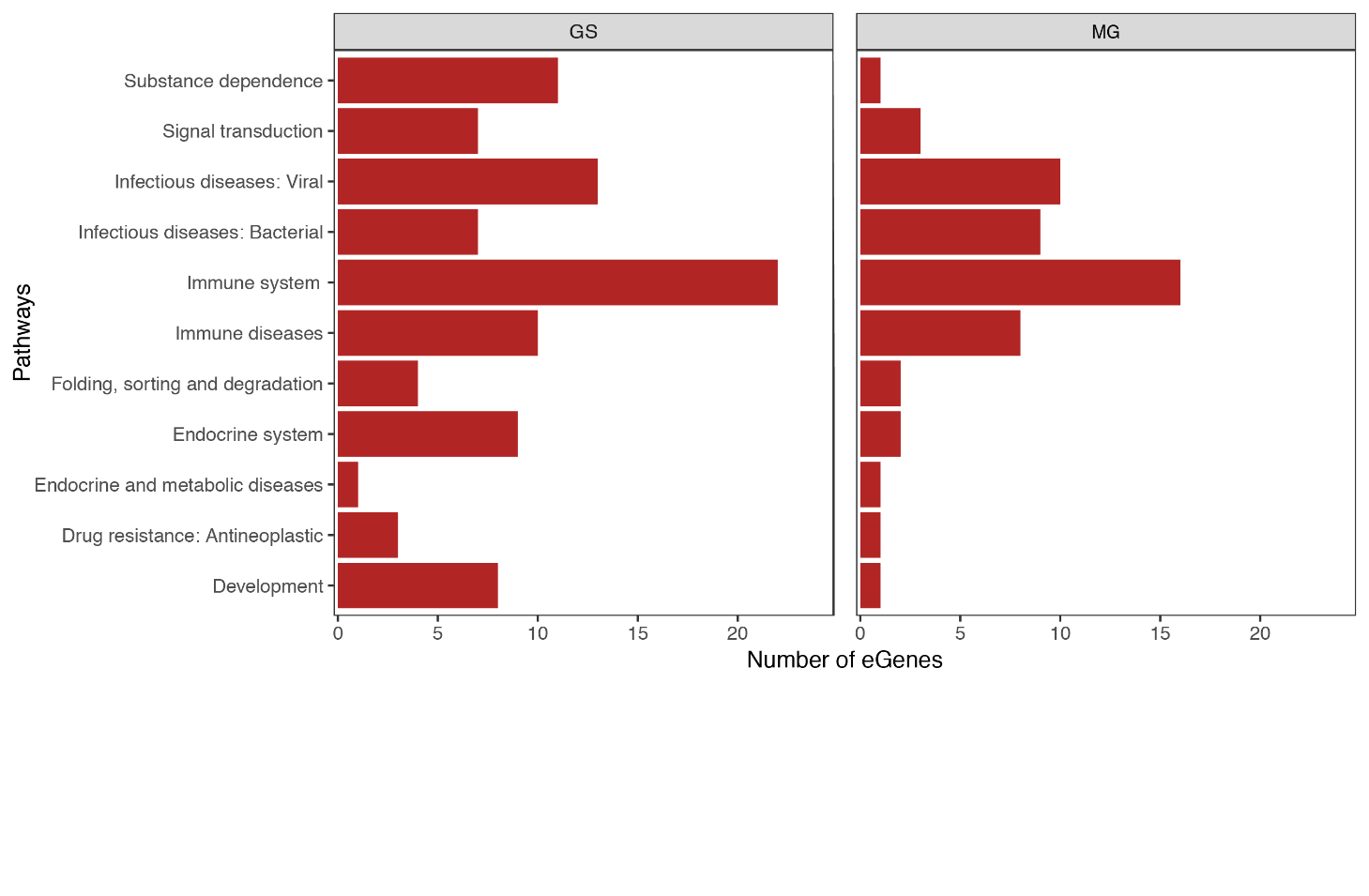
**

**Supplementary Figure 5.** GS and MG eGenes identified from immortalized cell lines share pathways. Twenty four pathways **(Supplementary Table 7a)** classified under these 11 categories (y-axis) were shared by GS and MG eGenes. Red bars indicate the number of eGenes participating in those pathways from GS (left) and MG (right).

**
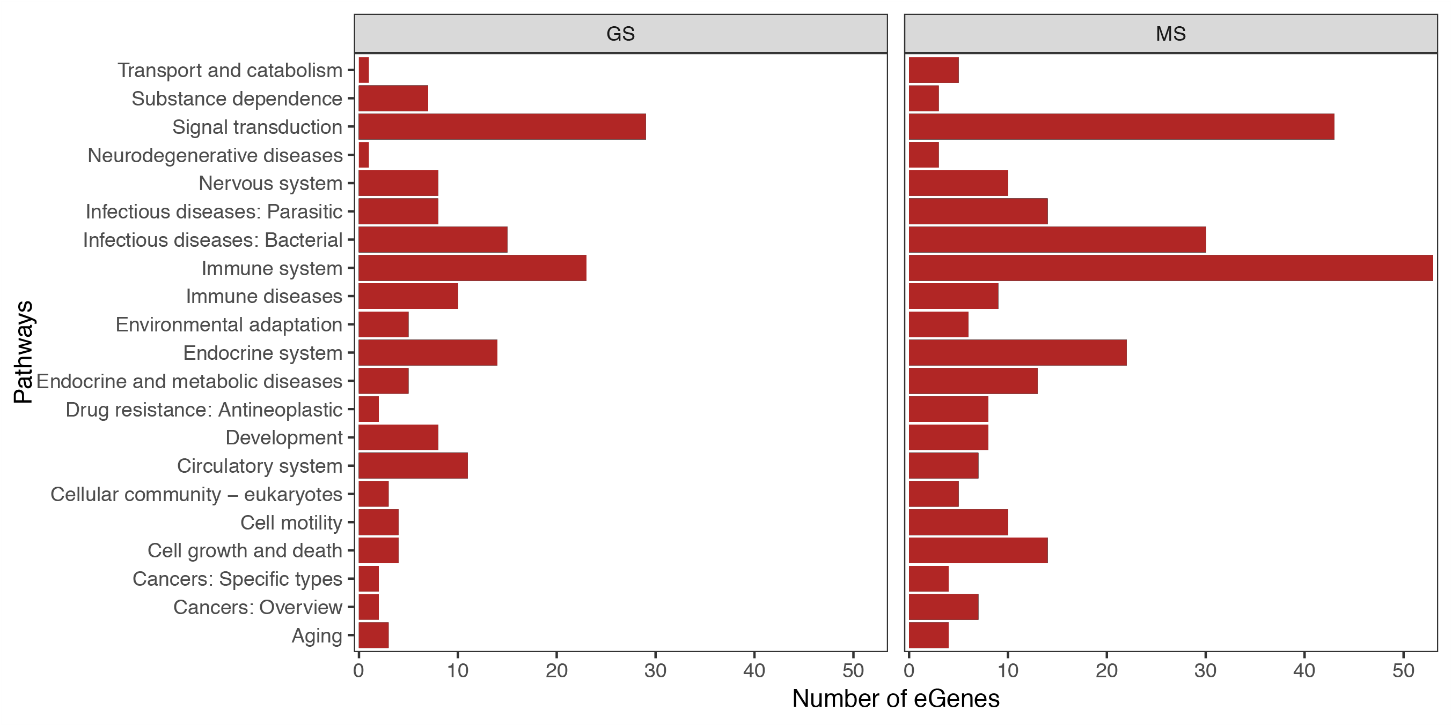
**

**Supplementary Figure 6.** GS and MS eGenes identified from immortalized cell line share pathways. Sixty nine pathways **(Supplementary Table 7b)** classified under these 21 categories (y-axis) were found to be shared by GS and MG eGenes. Red bars indicate the number of eGenes participating in those pathways from GS (left) and MS (right).

**
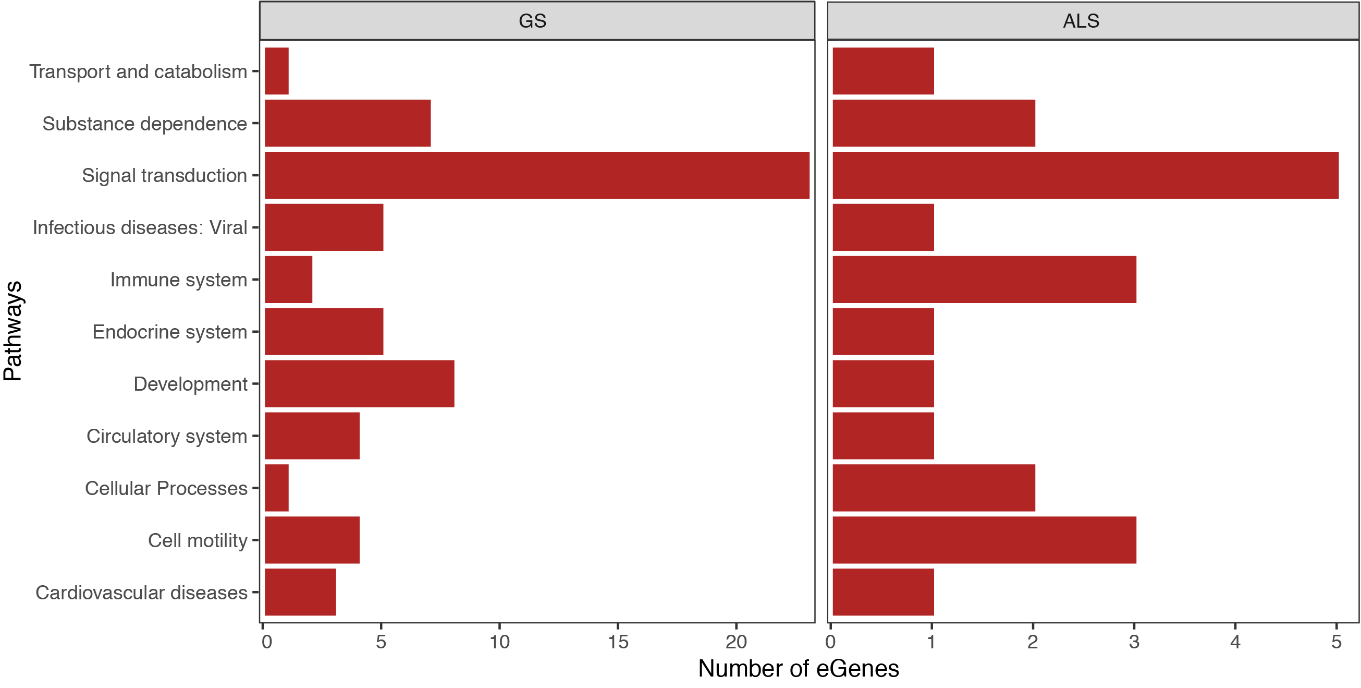
**

**Supplementary Figure 7.** GS and ALS eGenes identified from immortalized cell lines share pathways. Seventeen pathways **(Supplementary Table 7c)** classified under these 11 categories (y-axis) were shared by GS and ALS eGenes. Red bars indicate the number of eGenes participating in those pathways from GS (left) and ALS (right).


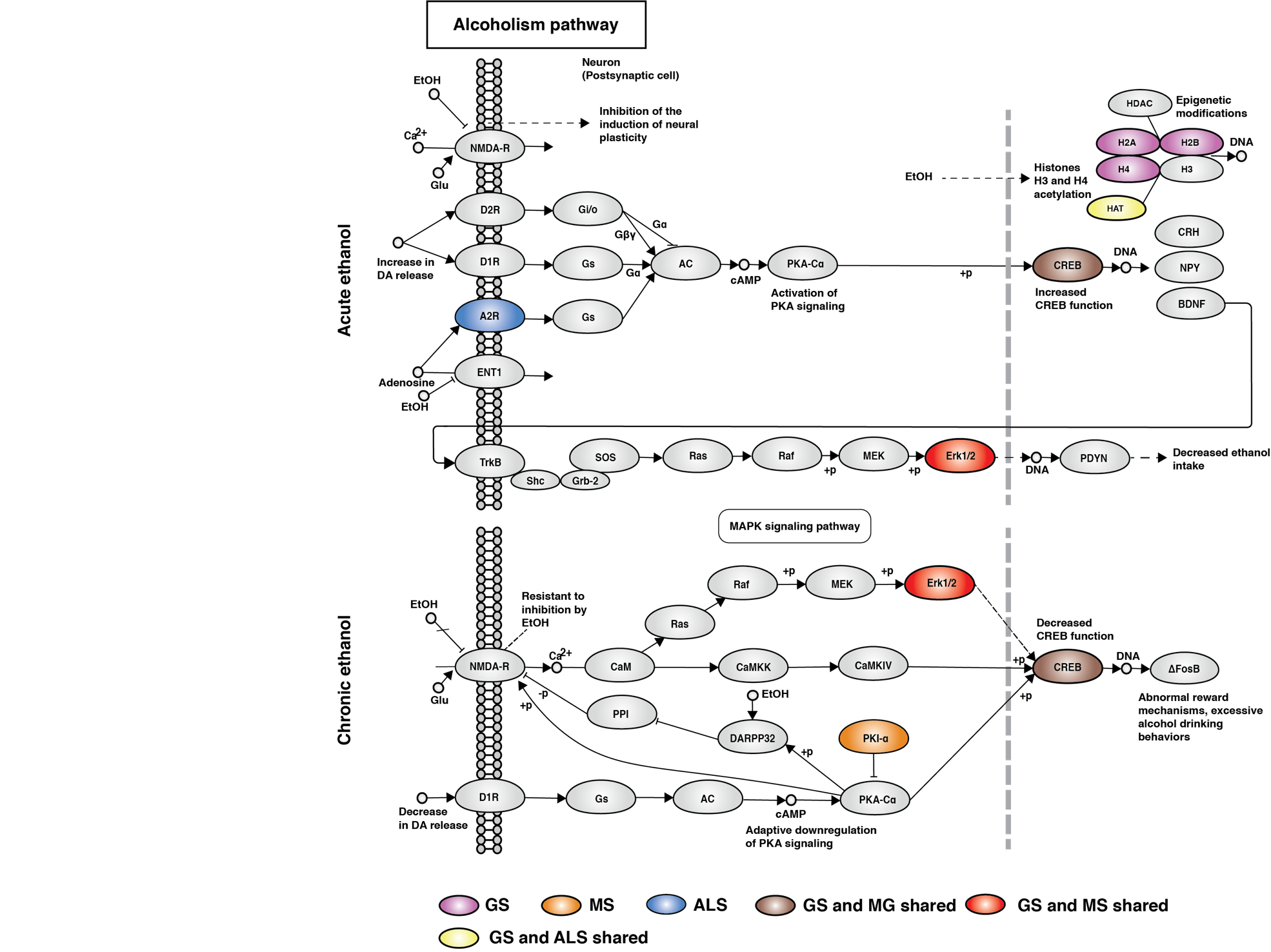


**Supplementary Figure 8.** eGenes from all four phenotypes (GS, MG, MS and ALS) co-occurred within the alcoholism pathway. Chronic exposure to alcohol leads to pathological changes in several tissues and organs. Alcohol primarily impairs global protein synthesis under basal conditions as well as in response to the anabolic stimuli including muscle contraction^58^. GS-, MG-, MS- and ALS-associated eQTLs spatially regulate several eGenes that encode proteins involved in both acute and chronic alcohol-mediated signaling.

**Supplementary Figure 9.** Pathway overlaps identified between all four phenotypes from both Hi-C datasets are statistically significant. Boxplots showing the mean and range of bootstrapping values (n=10000) for overlaps between randomly selected pathways (from a set of all pathways containing phenotype associated eGenes) to validate overlaps from the immortalized cell line Hi-C dataset **(**left**)** or the muscle Hi-C dataset **(**right**)**. The black point represents the observed number of pathway overlaps between the stated phenotypes. **** p-value < 1x10^-4^ ** p-value < 0.01 *p-value <0.05
